# Implementing Patient-Initiated Follow-Up (PIFU) Into a Psychiatric Outpatient Setting

**DOI:** 10.1192/bjo.2025.10525

**Published:** 2025-06-20

**Authors:** Thomas Scurr, Elizabeth Frayne, Isabelle Needham

**Affiliations:** 1 University of Exeter, Exeter, United Kingdom.; 2 Devon Partnership Trust, Exeter, United Kingdom

## Abstract

**Aims:** The cost to the NHS of missed appointments each year is highly significant. PIFU is an alternative to the conventional follow-up model where patients request appointments as-needed in attempt to reduce this cost, and is part of the Outpatient Recovery and Transformation Programme component of the NHS Long-Term Plan. This model is well established in chronic conditions under secondary care like gynaecology and rheumatology outpatients but has more recently been brought into psychiatry. Currently, there is minimal research on the suitability of this model in psychiatry. The Exmouth CMHT in Devon have had a PIFU model for the last 4 years, and this project evaluated this model and analysed the associated costs.

**Methods:** We present the model used to form the PIFU service in Exmouth. A service evaluation was conducted of the Exmouth PIFU model and is presented in this poster looking at the team constructed, pathways into the service, and the hours this service provided for patients. Patients under the service also have given feedback on their experience of the service. We then compare the costs of this service with equivalent referrals through primary care.

**Results:** In the absence of a standardised PIFU model for psychiatry, the Exmouth CMHT model was compared with the PIFU model described in the NHS Long-Term Plan. Our service evaluation demonstrates that limited staffing and budget can provide a suitable PIFU service for our patients. Patients gave positive feedback about their experience of PIFU and felt this had benefited their care. Cost comparisons demonstrate the relative costs, overall demonstrating savings to the NHS.

**Conclusion:** Despite a lack of research to guide the transition of PIFU into psychiatry, the Exmouth CMHT have created an effective model for their team that patients have found helpful. This model was adapted to the changing needs of the service over the years, demonstrating flexibility in the model, but despite this, it could be used as a template for the implementation of PIFU in other services. Cost comparisons demonstrate the saved time in primary care is most significant. Further research is planned to develop an evidence-based model for PIFU, and to look at staff perceptions of PIFU implementation.

